# Evaluating lung cancer screening disparities in an integrated healthcare system: barriers and opportunities

**DOI:** 10.3389/fonc.2025.1601458

**Published:** 2025-08-22

**Authors:** Carmen Javier, Sheng-Fang Jiang, Jenna Philippe, Isabel Arana, Jeffrey B. Velotta

**Affiliations:** ^1^ Internal Medicine, Kaiser Permanente San Francisco, San Francisco, CA, United States; ^2^ Division of Research, Kaiser Permanente Division of Research, Pleasanton, CA, United States; ^3^ School of Medicine, Kaiser Permanente Bernard J. Tyson School of Medicine, Pasadena, CA, United States; ^4^ Thoracic Surgery, Kaiser Permanente Oakland, Oakland, CA, United States

**Keywords:** lung cancer screening, low dose computed tomography (LDCT), LDCT lung cancer screening, integrated healthcare system, Kaiser Permanente, Northern California, health disparities, lung cancer

## Abstract

**Rationale:**

The national average rate of lung cancer screening (LCS) has remained low at roughly 6%, with California’s rate among the lowest at 1% compared to all fifty states.

**Methods:**

We enrolled Kaiser Permanente Northern California (KPNC) patients eligible for LCS per the USPSTF guidelines published in 2013 and 2021, respectively. Annual and overall rates of completed initial low-dose computed tomography of chest (LDCT) were computed from February 2015 to February 2022. Chi-squared tests and multivariable Cox regression assessed the impact of sociodemographic factors.

**Results:**

The average annual completion rate of initial lung cancer screening over the entire study period was 0.95% per the 2013 USPSTF guidelines. In the year 2022, only 0.69% of all eligible study participants per the 2021 USPSTF guidelines completed lung cancer screening. Chi-squared tests demonstrated differences in the overall proportion of individuals screened across the entire study period stratified by sex and race/ethnicity respectively (2013 USPSTF guidelines; 4.72% Males, 4.29% Females, p = 0.09 for the sex categories and Asian 4.31%, African American 3.89%, Hispanic 3.79%, Other 3.48%, Non-Hispanic White 4.79%, p = 0.02 for the race/ethnicity categories. Multivariate time-to-completion analyses demonstrated statistically significant associations for younger age groups (50-60: HR 1.41, 95% CI 1.21–1.64, p < 0.0001, 61-70: HR 1.95, 95% CI 1.68–2.27, p < 0.0001), male sex (HR 1.17, 95% CI 1.07–1.28, p = 0.0009), and all non-White racial/ethnic groups (Asian: HR 0.73, 95% CI 0.62–0.86, p = 0.0002, African American: HR 0.64, 95% CI 0.53–0.78, p < 0.0001, Hispanic: HR 0.66, 95% CI 0.55–0.80, p < 0.0001, Other: HR 0.75, 95% CI 0.60–0.93, p = 0.0086). Neighborhood Deprivation Index (NDI) quartiles were not significantly associated with initial LDCT completion (HRs 0.93 to 1.04; all p-values > 0.3).

**Conclusion:**

This average annual rate of LCS at KPNC was comparable to the statewide average in California. Age 61–70 years old, male sex, and non-Hispanic White race/ethnicity were the strongest and most statistically significant predictors of initial LDCT completion. NDI was not associated with screening uptake. No significant improvement in screening uptake was observed within the first year following the release of the 2021 USPSTF guidelines on LCS.

## 1 Introduction

Lung Cancer is the leading cause of cancer-related death in the United States and throughout the world. According to the American Cancer Society’s annual cancer statistics published in 2025, “lung cancer continues to dwarf other cancers in the number of deaths, causing more deaths in 2022 than colorectal, breast, and prostate cancers combined” (1). Globally, the World Health Organization has noted lung cancer as the “leading cause of cancer-related deaths worldwide” (2). Early detection of lung cancer is critical for improving survival outcomes. According to the Surveillance, Epidemiology, and End Results (SEER) database, the national, five-year survival rate from non-small cell and small cell lung cancers drop dramatically from early or localized stage (65% for non-small cell lung cancer, 30% for small cell lung cancer). to advanced or distant stage (9% for non-small cell lung cancer, 3% for small cell lung cancer) (3). Unfortunately, most lung cancers are diagnosed at an advanced stage when effective interventions are more limited (4). The recommendation of low dose computed tomography (LDCT) for lung cancer screening was first adopted by the United States Preventive Services Task Force (USPSTF) in 2015, after a groundbreaking publication in 2011 by the National Lung Screening Trial Research Team that revealed a 20.0% relative reduction in mortality from lung cancer with low-dose CT screening compared to radiography (5). However, to date, the national uptake of lung cancer screening per these recommendations is incredibly low. Every year, only about 5% of all eligible adults in the US considered at risk for lung cancer are screened (6, 7). That is a rate that is at least ten times lower than the annual, national screening rates for other cancers such as colorectal, cervical, or breast per reports by the American Cancer Society (8). Thus, the urgency to understand and address the challenges of lung cancer screening uptake could not be more pronounced.

Examining the role of sociodemographic and socioeconomic factors in the uptake of lung cancer screening has gained significant interest and momentum among various disciplines in the scientific community. In a secondary analysis of the National Lung Screening Trial (NLST) on racial differences in outcomes, non-Hispanic Black participants screened with LDCT experienced a greater reduction in lung-cancer specific mortality compared to non-Hispanic White (hazard ratio 0.61; 95% CI, 0.37 - 1.01 versus hazard ratio 0.86; 95% CI, 0.75-0.98) (9). The same study found that its non-Hispanic Black participants were younger, more likely to be current smokers, had more comorbidities, and had fewer years of formal education compared to its non-Hispanic White participants (p<0.05). Furthermore, a narrative review of multiple studies conducted in the US from 2010–2020 noted that higher income and higher education were not only associated with higher lung cancer screening rates, but also a higher likelihood of screening eligibility (10). Specifically, this narrative review found that smoking habits differed across income brackets. w Individuals with low income were more likely to start smoking at a younger age and smoke more heavily than those with higher income status, thereby developing high-risk for lung cancer at a younger age and with fewer pack-years (10). were In part to address these lung cancer screening disparities and from the findings of the NELSON trial, in 2021 the USPSTF updated its 2013 guidelines to include younger (the minimum age dropped from 55 to 50 years old) current and former smokers with less tobacco exposure (the minimum cigarette pack-years dropped from 30 to 20 pack-years) (7).

There is limited research to date on whether updating the USPSTF guidelines in 2021 had a positive effect on the prevalence of lung cancer screening. Kaiser Permanente Northern California is a large and nationally recognized integrated healthcare system serving a diverse patient population with an emphasis on preventive and comprehensive care, offering an opportunity to examine real-time changes within this setting. This study aimed to understand the state of lung cancer screening and identify the impact of sociodemographic factors on LCS completion with LDCT within a large integrated health system.

## 2 Methods

This was a retrospective cohort study comparing two groups of patients eligible for lung cancer screening, as defined by the 2013 and 2021 USPSTF guidelines, over a seven-year period (February 10^th^, 2015 – February 10^th^, 2022). The start date of this study period was chosen to match the launch date of KPNC’s lung cancer screening referral program and its integration with the electronic medical record. As a non-profit health maintenance organization recognized for its leadership in preventive and integrated care, KPNC provided a robust data collection and analysis platform encompassing a large, diverse, and stable membership base with minimal loss to follow-up (11). This was a data-only cohort study with strict adherence to HIPAA standards. Approval for this study was obtained from an independent Institutional Review Board.

### 2.1 Selection and description of participants

This study used the USPSTF recommendations for lung cancer screening published in 2013 and 2021 to define the eligibility criteria for its study participants (6). The inclusion criteria were adult KPNC patients who qualified for initial lung cancer screening according to USPSTF guidelines published in 2013 and 2021 respectively and had a minimum of 6 months continuous KPNC membership after self-reporting smoking status. Individuals with a diagnosis of lung cancer were excluded from this study.

In accordance with the 2013 USPSTF guidelines for lung cancer screening, we identified all adults 55 to 80 years old with a 30 pack-year cigarette smoking history and who either a) currently smoked or b) quit smoking within the last 15 years during the study period. Similarly, in accordance with the 2021 USPSTF guidelines for lung cancer screening, we identified all adults 50 to 80 years old with a 20 pack-year cigarette smoking history and who either a) currently smoked, or b) quit smoking within the last 15 years during the study period. Given that the 2021 guidelines expanded eligibility criteria, we expected the cohort selected using the 2021 guidelines to be larger in size and include all of the participants captured in the cohort selected using the 2013 guidelines. Only documentation of self-reported smoking history recorded by provider or medical assistant during patient visits within the electronic medical record was used to determine participants’ smoking status and cigarette pack years. We chart reviewed a random sample of ten percent of our study population to verify the accuracy of the smoking history data collected via coding analysis. In addition, to further verify accurate smoking information, we leveraged data from our KPNC Member Health Surveys to assess the reliability of smoking history data documented in the electronic medical record. Since 1993, the KPNC Member Health Survey has been conducted by mail triennially on independent age- and sex-stratified random samples of English-speaking, adult, KPNC members (12, 13).

The sociodemographic factors selected for this study included age, biological sex, race/ethnicity, and neighborhood deprivation index. The race/ethnicity categories analyzed in this study were Asian, African American, Hispanic, Non-Hispanic White, and Other. We used the names and definitions for each race/ethnicity category in accordance with reporting by the US Office of Management and Budget as well as the US Bureau of the Census (14). Due to an insufficient sample size of participants who identified as Pacific Islander, Native American, or multi-racial, these individuals were grouped into the category named “Other”. There were eight socioeconomic variables that contributed to the neighborhood deprivation index: percent of households earning below the federal poverty level, percent of households earning less than $30,000 per year estimating poverty, percent of households on public assistance, percent of individuals unemployed, percent of males in management and professional occupations, percent of female-headed households with dependents, percent of crowded housing, and percent of individuals earning less than a high school diploma. These variables were adopted from Messer et. al.’s validated principal component analysis model for building a neighborhood deprivation index using comprehensive US census-tract level data, in-depth literature review of the social determinants of health, and a stepwise statistical approach to construct an index with stability and generalizability across diverse populations (15).

### 2.2 Data collection and measurements

Non-identifiable patient data was collected through the electronic health record at Kaiser Permanente Northern California. The names of the databases used were Clarity, DOR Research Database, and Virtual Data Warehouse (VDW). Current Procedural Terminology (CPT) codes 02095 (Computed Tomography of Thorax, Low Dose Screening) and 71271 (Computed Tomography, Thorax, Low Dose for Lung Cancer Screening, Without Contrast Material(S)) were used to identify low-dose chest tomography (CT) scans completed for lung cancer screening during the entire study period. There were no other CPT codes used within this closed health system to identify LDCT scans indicated for lung cancer screening. In addition to CPT codes, we also searched for a specific text string, “#LCS%” in the CT reports. This text string is part of an internal system used for tagging CT scans ordered specifically for lung cancer screening. Using both the CPT codes and the text string tag ensured accuracy and reliability in our data collection process.

### 2.3 Statistics

All statistical analyses for this study were performed using Statistical Analysis Systems (SAS), Version 9.4 software. Statistical significance was set at 0.05. The proportions of study participants in each cohort who completed initial LDCT for lung cancer screening across time during our study period was used to compute rates. Chi-squared tests were used to compare these rates by sex and by race/ethnicity respectively.

A Cox proportional hazards regression analysis was used to evaluate univariate and multivariate relationships between sociodemographic factors and time-to-completion of initial LDCT. Person-time contribution was measured with start time defined as the date when each participant became eligible for LCS per the 2021 USPSTF guidelines. Participants were followed from study entry until they either completed LDCT, were censored (due to lost to follow-up, death, or disenrollment from the Kaiser Permanente Health plan), or reached the end date of the study period, whichever occurred first. Univariate and multivariate hazard ratios (HRs) were estimated by comparing covariates of those who had the event to those remaining in the risk set through the partial likelihood function in Cox regression. Corresponding 95% confidence intervals (CIs) and p-values were computed.

The sociodemographic categories analyzed in the cox regression model included age, sex, race/ethnicity, and neighborhood deprivation index (NDI). The NDI computed for this study was adapted from Messer et. al.’s principal component analysis (PCA) model (15). In their study, Messer et al. identified eight out of twenty components with the highest, stable, and consistent loadings (0.2 -0.4) observed within individual regions, across regions, and in the combined all-site analysis of regions representing eight geographically diverse areas (15). The resulting first principal component was chosen as the NDI and accounted for 51 to 73% of the total variability in the component measures (15). Loading values from the established PCA model were applied to weight each standardized variable in our data, and the weighted variables were summed to produce the NDI score. Scores were categorized into quartiles, Quartile 1 through Quartile 4, such that the highest quartile represented the most neighborhood deprivation.

## 3 Results

### 3.1 Cohort demographics and overall screening uptake

**Table 1** provides the distribution of the demographic characteristics (age group, sex, and race/ethnicity) for our study participants grouped by lung cancer screening guideline used for enrollment. During the entire study period from February 2015 thru February 2022, a total of 28,046 patients were identified as eligible for lung cancer screening (LCS) under the 2013 USPSTF guidelines, while 60,676 patients were identified as eligible under the 2021 USPSTF guidelines, a more than twofold increase in the eligible population. Overall, the distributions for sex and race/ethnicity were similar among both study groups but the distribution of age differed, with the largest age group shifting to the youngest age category (≤60 years old) under the 2021 LCS guidelines.

**Table 1:** Demographic characteristics of study participants.

Demographic Characteristic	Cohort per USPSTF 2013 LCS Guidelines, N (%)	Cohort per USPSTF 2021 LCS Guidelines, N (%)
Age (years)		
≤60*	9087 (32.4)	28537 (47.0)
61-70	13233 (47.2)	22540 (37.2)
71-80	5726 (20.4)	9599 (15.8)
Sex		
Female	10997 (39.2)	25090 (41.4)
Male	17049 (60.8)	35586 (58.6)
Race/Ethnicity		
Asian	2273 (8.1)	5823 (9.6)
Black	1900 (6.8)	4705 (7.8)
Hispanic	1980 (7.1)	4871 (8.0)
Other	1437 (5.1)	3203 (5.3)
White	20456 (72.9)	42074 (69.3)
**Total**	**28,046**	**60,676**

USPSTF, United States Preventive Services Task Force; LCS, Lung Cancer Screening.

* The youngest age eligible differed by guideline used. For the USPSTF 2013 guidelines, the youngest age eligible was 55 years old. For the USPSTF 2021 guidelines, the youngest age eligible was 50 years old.

– – – – – –

Overall, 1,277 (4.55%) of the study participants eligible for initial lung cancer screening per the 2013 USPSTF guidelines completed screening during the entire study period. Because only the final year of the study period (year 2022) reflects eligibility under the 2021 USPSTF guidelines, we did not calculate an overall screening proportion for the entire study period under these updated guidelines. The attrition rate for this study was 14.8%.

### 3.2 Annual lung cancer screening rates

**Figure 1:**
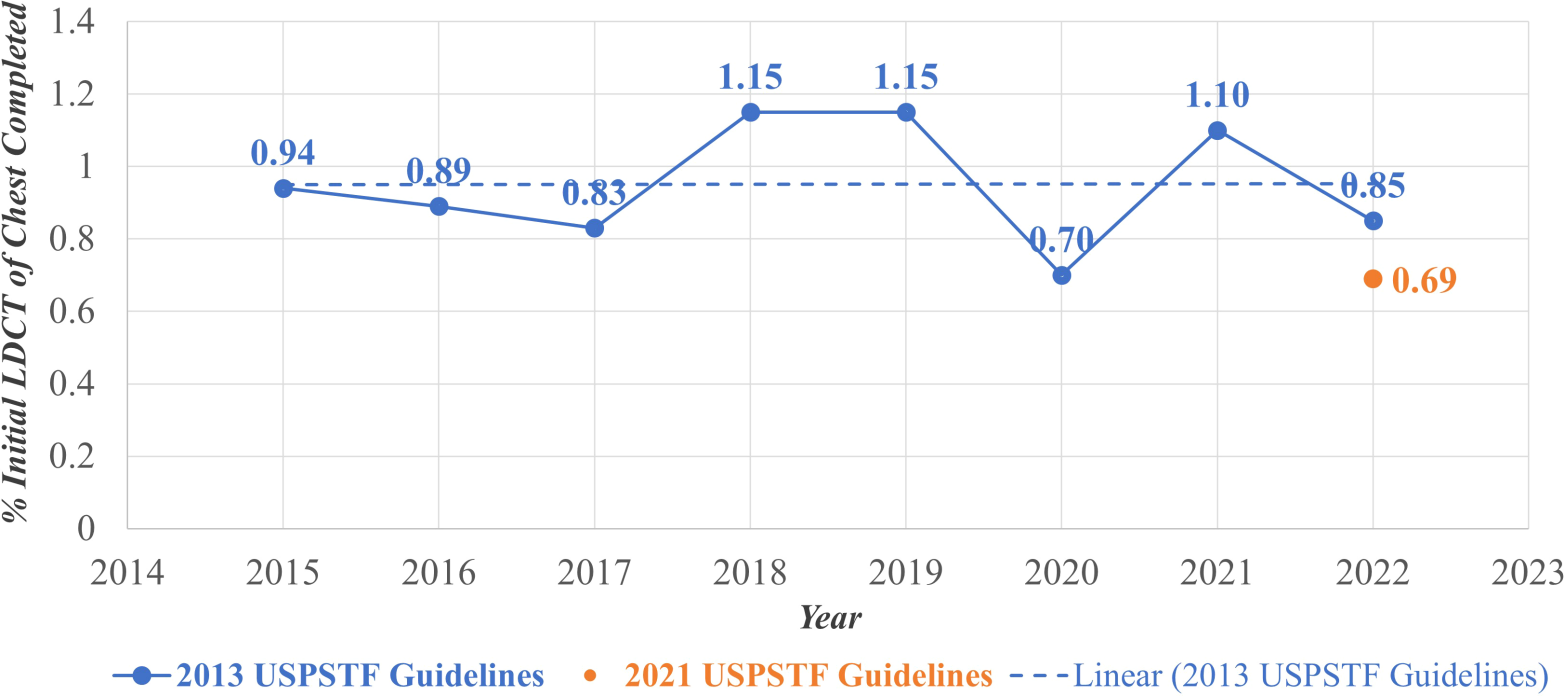
Annual lung cancer pptake, February 2015-February 2022. LDCT, Low Dose Computed Tomography; USPSTF, United States Preventive Services Task Force.

**Figure 1** displays a scatterplot of the annual percentages of initial LDCT for lung cancer screening completed in each year of the study period. The average annual percentage of lung cancer screening uptake across the seven-year study period was 0.95% using the 2013 USPSTF guidelines. This average is represented by the dashed line in **Figure 1**. The highest annual uptake was observed in the years 2018 and 2019, with a completion rate of 1.15% in each year. The year 2020 demonstrated a stark decrease in screening uptake, with only a 0.70% completion rate. In the year 2022, roughly a year since the 2021 USPSTF guidelines were activated, the completion rate among those eligible per these guidelines was 0.69%. By comparison, using the 2013 USPSTF guidelines to determine eligibility in the year 2022 resulted in a 0.85% completion rate.

### 3.3 Overall lung cancer screening uptake stratified by sex

**Figure 2:**
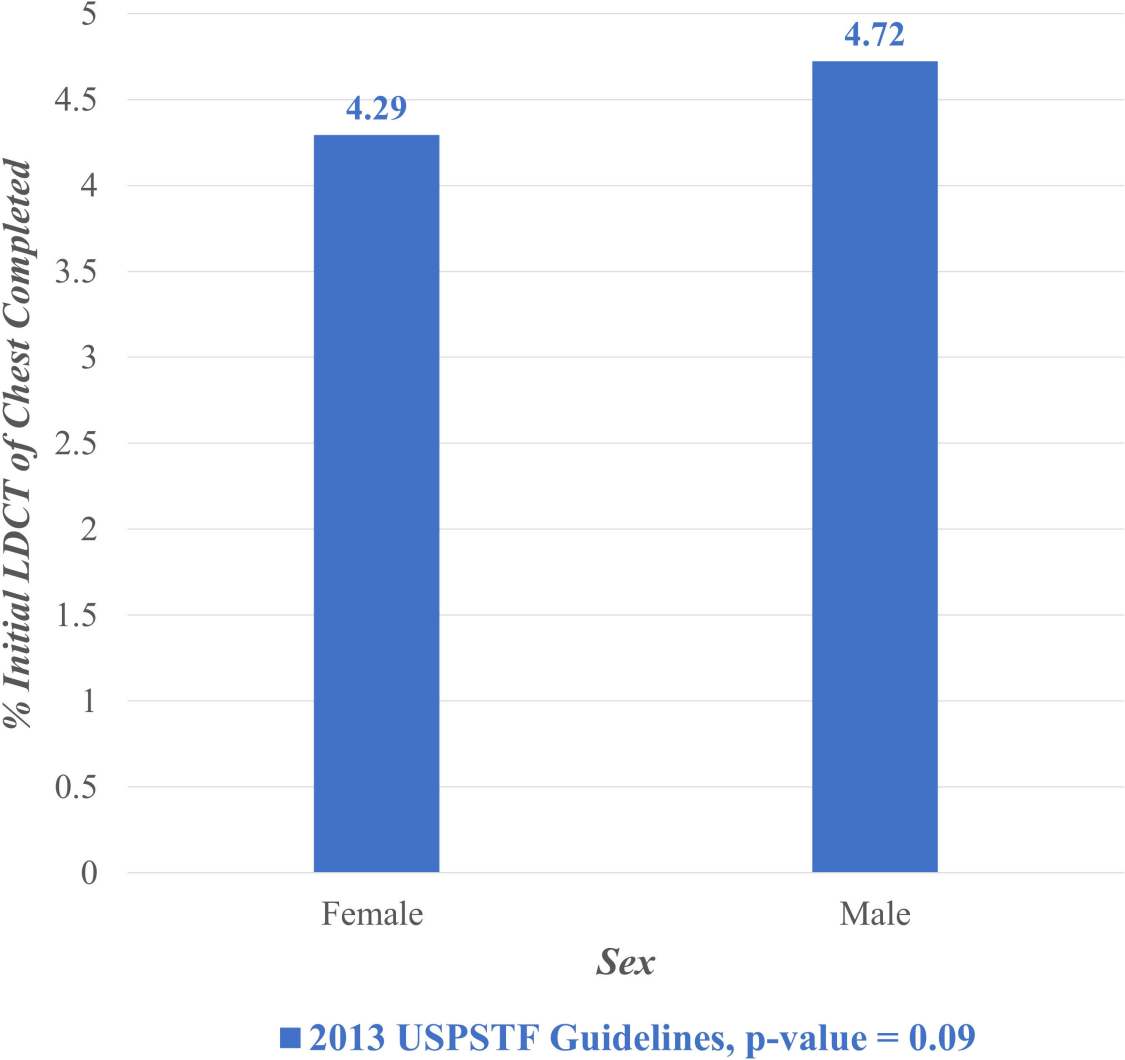
Lung cancer screening stratified by sex, February 2015- February 2022. LDCT, Low Dose Computed Tomography; USPSTF, United States Preventive Services Task Force.

**Figure 2** displays the proportion of eligible individuals who completed initial LDCT lung cancer screening during the entire study period (February 2015 – February 2022) stratified by sex. Among eligible individuals per the USPSTF 2013 guidelines, 4.72% of males completed screening compared to 4.29% of females. A chi-squared test was performed to compare these two proportions and found it not to be statistically significant (p = 0.09).

### 3.4 Overall lung cancer screening uptake stratified by race/ethnicity

**Figure 3:**
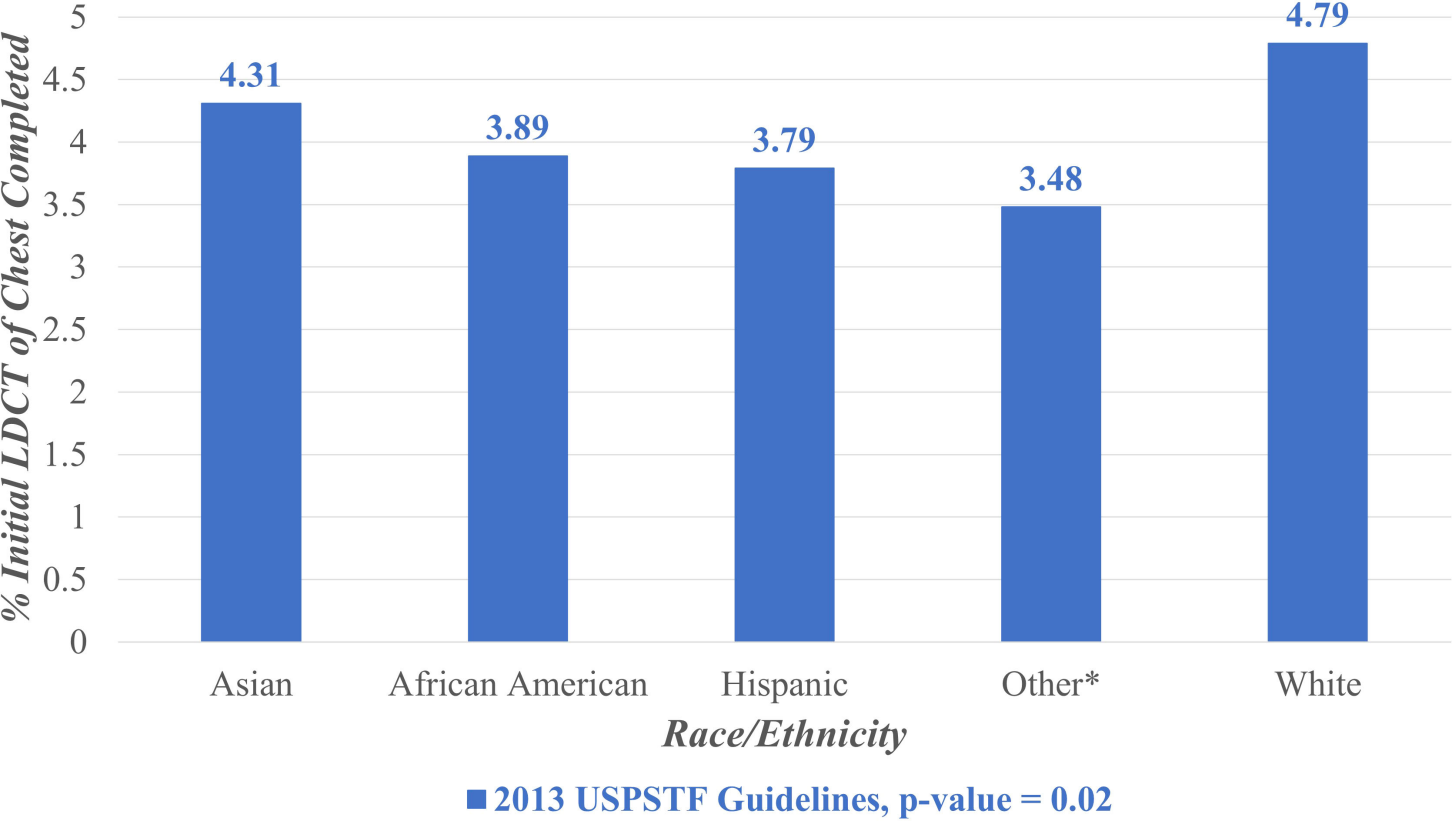
Lung cancer screening stratified by race/ rthnicity, February 2015- February 2022. LDCT, Low Dose Computed Tomography; USPSTF, United States Preventive Services Task Force. *Other includes Pacific Islander, Native American, and multi-racial minority groups.

**Figure 3** displays the proportion of individuals who completed initial LDCT lung cancer screening during the entire study period (February 2015 – February 2022) stratified by race/ethnicity. Under the 2013 guidelines, completion rates were highest among non-Hispanic White individuals (4.79%), followed by Asian (4.31%), African American (3.89%), Hispanic (3.79%), and Other racial/ethnic groups (3.48%). The Other racial/ethnic category encompassed Pacific Islander, Native American, and multi-racial minority groups.

A chi-squared test was performed to assess differences in the overall proportions screened by racial/ethnic group and revealed a statistically significant difference (p = 0.02).

### 3.5 Sociodemographic predictors of lung cancer screening

**Table 2** presents the estimated hazard ratios (HRs), 95% confidence intervals (CIs), and p-values for sociodemographic factors in a cox proportional hazards analysis of time-to-completion of initial LDCT. Compared to participants aged 71–80 years, those in younger age groups demonstrated a significantly higher likelihood of completing initial lung cancer screening with LDCT. In the multivariate analysis, participants aged 50–60 years had a 41% higher hazard of screening completion (HR 1.41; 95% CI 1.21–1.64; p < 0.0001), and those aged 61–70 years had a two-fold higher hazard (HR 2.00; 95% CI 1.68–2.27; p < 0.0001). Male participants were also more likely to complete LDCT screening compared to females, with a 17% higher hazard in the multivariate analysis (HR 1.17; 95% CI 1.07–1.28; p = 0.0009). Across racial and ethnic groups, all non-White participants had a lower likelihood of completing LDCT screening compared to non-Hispanic White participants. In the multivariate analysis, Asian participants had a 27% lower hazard (HR 0.73; 95% CI 0.62–0.86; p = 0.0002), African American participants had a 36% lower hazard (HR 0.64; 95% CI 0.53–0.78; p < 0.0001), Hispanic participants had a 34% lower hazard (HR 0.66; 95% CI 0.55–0.80; p < 0.0001), and participants classified as “Other” had a 25% lower hazard (HR 0.75; 95% CI 0.60–0.93; p = 0.0086) compared to non-Hispanic Whites.

**Table 2:** Sociodemographic predictors of time to initial low-dose CT screening completion.

Covariates	Unadjusted HR	95% CI	P-value	Adjusted HR	95% CI	P-value
Age group						
50-60	1.39	1.19 - 1.62	<0.001	1.41	1.21 - 1.64	<0.001
61-70	1.94	1.67 - 2.30	<0.001	2.0	1.68 - 2.27	<0.001
71-80	1.00 (ref)			1.00 (ref)		
Sex						
Male	1.13	1.04 - 1.24	0.007	1.17	1.07 - 1.28	0.001
Female	1.00 (ref)			1.0 (ref)		
Race/ethnicity						
Asian	0.77	0.65 - 0.91	0.002	0.73	0.62 - 0.86	<0.001
African American	0.67	0.55 - 0.81	<0.001	0.64	0.53 - 0.78	<0.001
Hispanic	0.68	0.56 - 0.82	<0.001	0.66	0.55 - 0.80	<0.001
Other	0.75	0.60 - 0.93	0.010	0.75	0.60 - 0.93	0.009
White	1.00 (ref)			1.00 (ref)		
Neighborhood Deprivation Index						
Quartile 1	1.0 (ref)			1.00 (ref)		
Quartile 2	0.92	0.79 - 1.07	0.268	0.93	0.80 - 1.07	0.300
Quartile 3	0.94	0.81 - 1.08	0.372	0.95	0.82 - 1.10	0.497
Quartile 4	0.97	0.83 - 1.13	0.676	1.04	0.89 - 1.21	0.626
Unknown*	0.93	0.30 - 2.90	0.900	0.96	0.31 - 3.00	0.947

CT, Computed Tomography; HR, Hazard Ratio; CI, Confidence Interval.

*Unknown represents less than 0.2% of study participants for whom a neighborhood deprivation index could not be derived due to missing information (ex: missing zip code).

Univariate and Multivariate hazard ratios with corresponding 95% confidence intervals and p-values for sociodemographic factors age, sex, race/ethnicity and neighborhood deprivation index (NDI).

Quartile 1 represents the least deprived neighborhoods, while quartile 4 represents the most deprived neighborhoods.

There were no statistically significant differences in LDCT screening completion across neighborhood deprivation index (NDI) quartiles in either univariate or multivariate analyses (See **Table 2**). For example, participants in NDI quartile 4 had an adjusted HR of 1.04 (95% CI 0.89–1.21; p = 0.6258) compared to those in quartile 1. Univariate analyses of each of the aforementioned sociodemographic categories examined in this Cox regression revealed statistically significant associations with time-to-completion of LDCT screening (see **Table 2**) that remained consistent in direction and magnitude as their corresponding multivariate analyses, suggesting minimal confounding by other sociodemographic variables. Given the large sample size and the narrow confidence intervals observed for the hazard ratio estimates, we focused our reporting on these effect sizes and their precision, rather than on the number of events by covariate category.

## 4 Discussion

This retrospective study reflects the state of lung cancer screening in routine clinical practice within a large integrated healthcare system from 2015 through 2022. Under the 2013 USPSTF guidelines, the average annual rate of lung cancer screening in our study was 0.95%, modestly higher than the estimated 0.7% annual rate for California by the American Lung Association’s 2022 State of Lung Cancer Report (2), representing a relative difference of approximately 39%. With the exception of 2020, the annual rate of initial lung cancer screening in the 2013 guideline cohort remained above 1% from 2018 onward during the study period. This suggests a minimum three-year interval following the implementation of the lung cancer screening referral program at Kaiser Permanente Northern California before achieving and exceeding the state’s annual screening rate. The wake of the COVID-19 pandemic likely influenced the steep decline in lung cancer screening rate to 0.7% in 2020. According to a survey of 116 lung cancer screening programs in the GO2 Foundation Centers of Excellence in Lung Cancer Screening network across the United States, 56% of screening programs reported moderate to severe decreases in new patient volume during this same time period (16). This stark reduction in screening during the COVID-19 pandemic likely led to delayed diagnosis and highlights the need for more innovative modes (ex: blood, nasal specimens) of screening for lung cancer that would mitigate the demand for tertiary healthcare services such as CT imaging.

Notably, the annual screening proportions were lower than the overall screening proportion observed across the entire study period. This discrepancy reflects the difference between year-specific uptake and cumulative screening completion. Annual proportions capture individuals screened within a single calendar year (ex: **Figure 1**), while the overall proportion reflects individuals who completed screening at any point during the seven-year study period (ex: **Figures 2**, **3**). Because individuals may be screened in different years, the cumulative measure aggregates across all years and therefore appears higher than any single year’s uptake. Additionally, temporal factors such as evolving guideline implementation may have contributed to year-to-year variability in screening rates.

The expanded eligibility for lung cancer screening under the updated USPSTF guidelines in March 2021 did not result in an increase in screening uptake during nearly one year of applicable follow-up in our study period (February 10^th^, 2015 – February 10^th^, 2022). While the impetus for the expansion of LCS eligibility was multi-faceted, reducing disparities in screening toward racial/ethnic minority groups and females were notable, evidence-based motives (17). The lack of an observed increase in screening rates suggests that broader eligibility criteria, while necessary, are insufficient on their own to improve screening uptake. Our findings point to an opportunity to address systemic barriers through targeted interventions that may enhance engagement and facilitate access to lung cancer screening in newly eligible populations. For example, establishing a patient navigator program that targets patients from underserved communities by assisting with health education, scheduling, and health insurance benefits clarification, may help improve screening completion rates and adherence to follow-up. Lay navigators can also help bridge language and cultural barriers as well as address historical mistrust in the healthcare system (18). We recently conducted a prospective case control study among Asian American patients of the Kaiser Permanente Northern California health system and found that active outreach via a lay patient navigator resulted in significantly higher lung cancer screening rates compared to passive outreach via email. These preliminary results are part of a pilot study that is currently under peer review for publication.

In 2022—the first full year following the publication of the updated USPSTF guidelines—the annual lung cancer screening rate among individuals eligible under the 2021 guideline criteria fell below the state rate, with only 0.69% of the cohort completing screening. This suggests a delay in the adoption of lung cancer screening guidelines into clinical practice even within an integrated healthcare system. While there are several considerations for this backlog, fostering clinician awareness and motivation for lung cancer screening might hold the greatest impact (19). In a knowledge assessment on lung cancer screening criteria given to 32 providers practicing primary care and pulmonology in Southern California, none correctly identified all of the eligibility criteria (20). Similarly, a cross-sectional study of 625 providers at Vanderbilt University found that not only was referring provider knowledge of lung cancer screening guidelines low, but it was also directly proportional to the ordering rate of LDCT for eligible patients (21). Timely and widespread training provided not only to physicians but to all the individuals involved (ex: patients, nurses, technicians, etc.) in the lung cancer screening and referral process may serve as a catalyst for significant improvement in uptake. Furthermore, leveraging technology to embed algorithms within the electronic healthcare record to facilitate the identification of eligible individuals for lung cancer screening can help relieve and perhaps motivate already strained medical personnel to offer this screening service to patients. A notable barrier not unique to the electronic medical record at Kaiser Permanente Northern California is effective notification/alert of patients eligible for lung cancer screening and/or updated smoking history.

While the chi-squared analysis demonstrated no statistically significant difference in the overall proportion of lung cancer screening completion between males and females, the adjusted Cox proportional hazards analysis revealed a statistically significant difference in the time-to-completion of initial screening. Specifically, males were more likely to complete screening earlier than females, after adjusting for age, race/ethnicity, and neighborhood deprivation index (NDI). These findings suggest that although the overall screening rates may appear similar, underlying disparities in timely access to screening exist. In a nationally representative survey conducted by the American Lung Association in 2022 to examine awareness, attitudes and beliefs of 4,000 Americans, only 10% of adults believed that lung cancer is among the most likely cancers to affect women while 35% believed that it was among those most likely to affect men (22). Targeted strategies to raise awareness and encourage timely screening completion among women may help address this disparity (22, 23).

Similarly, participants of every other race/ethnicity studied exhibited a lower rate of initial lung cancer screening compared to Non-Hispanic Whites, with the lowest rate observed for African American participants. Collaborating with community leaders and community organizations to foster effective targeted recruitment strategies, such as the Jefferson Lung Cancer Learning Community in Philadelphia, is an evidence-based method for ameliorating health disparities (24, 25).

Younger eligible patients aged 51–70 years old were more likely to complete initial screening for lung cancer compared to 71–80 years old. Given that lung cancer has a generally poor prognosis with an overall five-year survival rate of 26.7%, identifying this disease at a younger age may allow more treatment options to be available (26). In addition, there was no significant relationship between lung cancer screening uptake and neighborhood deprivation index. We believe this is attributable to the KPNC integrated health model which allows all members regardless of their socioeconomic background access to lung cancer screening at no additional costs. The relative stability of patient membership within Kaiser Permanente helps buffer against socioeconomic barriers to screening by ensuring continuity of care, minimizing disruptions in insurance coverage, and facilitating long-term engagement with preventive services. This integrated model allows patients, including those from lower socioeconomic backgrounds, to remain connected to the healthcare system over time, increasing opportunities for screening recommendations, outreach, and follow-up. Additionally, standardized workflows and embedded reminders across care settings reduce dependence on individual resources or health literacy, helping to equalize access to lung cancer screening. With the delay seen in CMS financial reimbursement for lung cancer screening under the updated 2021 USPSTF guidelines until February 2022 (27), further research beyond our study period may provide insight on the impact of this policy in promoting screening uptake within other healthcare models.

While this study provided valuable insights, there are some limitations that must be acknowledged. First, we will discuss design-specific limitations. The end date of the study period only included one year out of the seven-year study period in which the updated 2021 USPSTF guidelines were in effect. Hence, it is difficult to compare the long-term impact of both the 2013 and 2021 guidelines without an even distribution of years in which these guidelines were in effect. The end date selected for the study period was current at the time this research study received approval by the Institutional Review Board. Another limitation of this study includes that the primary study outcome only captured initial lung cancer screening, not routine follow-up lung cancer screening. National and statewide rates of lung cancer screening reported by organizations such as the American Lung Association and the American Cancer Society do not distinguish between initial and follow-up screenings estimated by the American Lung Association and American Cancer Society do not distinguish initial from follow-up screenings. To our knowledge, there is currently no validated registry or organization systematically tracking US rates of initial lung cancer alone. We chose to focus on initial lung cancer screening as this was an unexplored area of research within this healthcare system. Sakoda and colleagues have published previously on follow-up lung cancer screening within the same integrated healthcare system (28, 29). Next, smoking history data was obtained through self-reporting by patient, which may have resulted in some inaccurate or underreported smoking history due to recall bias and social desirability bias. However, in the context of the KPNC integrated healthcare system—where very few patients are lost to follow-up, the majority of patients are long-term health plan members, and documentation of smoking history is a mandated component of both ambulatory and inpatient workflows—there is less opportunity for incomplete or unreliable smoking histories.

Furthermore, there were also limitations not particularly unique to our study that should be highlighted. First, we used a Cox proportional hazards model which assumes proportional hazards over time. This means that the relative hazard between two groups stays constant across the entire study period even though the absolute risk may change over time. We used time-on-study as the underlying time scale for our cox regression, which may not account for secular trends (ex: the COVID-19 pandemic). We assumed independent censoring, meaning that the reason(s) a participant was censored was unrelated to their probability of completing screening or developing lung cancer. Although we adjusted for key sociodemographic factors, unmeasured confounding from clinical or behavioral factors may still influence the observed associations. Finally, the generalizability of these findings may be limited to similar integrated healthcare systems with established referral workflows and minimal loss to follow-up.

## 5 Conclusion

In conclusion, this retrospective study examined the state of initial lung cancer screening (LCS) in routine clinical practice within a large integrated healthcare system from 2015 through 2022. The average annual rate of initial LCS was 0.95% under the 2013 USPSTF guidelines and 0.66% under the 2021 USPSTF guidelines during the study period. Younger patients were more likely to undergo initial lung cancer screening, increasing the potential for earlier detection and improved prognosis if diagnosed at an earlier cancer stage. Male sex and non-Hispanic White race were also associated with significantly higher LCS rates and a greater likelihood of completing low dose CT screening compared to other sex and racial/ethnic groups. Socioeconomic status, as measured by neighborhood deprivation index (NDI) was not associated with time-to-completion of LDCT.

Although this study captured roughly one year’s worth of initial LCS data since the release of the 2021 USPSTF guidelines, significant disparities in screening uptake for racial/ethnic minorities and women within an integrated health care system were observed. While the 2021 USPSTF guidelines were designed to improve equity in LCS for these populations in particular, further efforts are needed within healthcare systems to fully realize this potential and achieve sustainable, long-term improvements in lung cancer prevention and outcomes.

## Data Availability

The raw data supporting the conclusions of this article will be made available by the authors, without undue reservation.
